# Chemical Inhibition of NRF2 Transcriptional Activity Influences Colon Function and Oestrogen Receptor Expression in Mice at Different Ages

**DOI:** 10.3390/ijms252413647

**Published:** 2024-12-20

**Authors:** Aleksandra Piechota-Polanczyk, Zanya Mariwani, Jakub Fichna, Andrzej Polanczyk, Alicja Jozkowicz

**Affiliations:** 1 Department of Cell Cultures and Genomic Analysis, Medical University of Lodz, 90-752 Lodz, Poland; 2 Department of Medical Biotechnology, Faculty of Biochemistry, Biophysics and Biotechnology, Jagiellonian University, Gronostajowa 7, 30-387 Krakow, Poland; zanya.mariwani@student.uj.edu.pl (Z.M.); alicja.jozkowicz@uj.edu.pl (A.J.); 3 Department of Biochemistry, Faculty of Medicine, Medical University of Lodz, Mazowiecka 5, 92-215 Lodz, Poland; jakub.fichna@umed.lodz.pl; 4 The Faculty of Safety Engineering and Civil Protection, Fire University, 01-629 Warsaw, Poland; apolanczyk@apoz.edu.pl

**Keywords:** NRF2, colon, oestrogen receptors, ML385

## Abstract

We aim to investigate whether chemical inhibition of NRF2 transcriptional activity (TA) influences distal colon contractions, particularly in an age-dependent manner in females, and whether it impacts oestrogen receptor signalling in female mice. This study was performed on 3 and 6-month-old female mice treated with ML385 (30 mg/kg) or a vehicle for 7 days (i.p.). The colon functionality was verified with a colon bead expulsion test; serum samples were collected for oestradiol levels, and colon samples were stored for various histological analyses. The results show that the seven-day treatment of ML385 significantly downregulated TA (*p* < 0.05) and impacted its contractility. Additionally, young females treated with ML385 exhibited an increase in goblet cell number and significantly increased ERα, but not ERβ, especially in older mice. It is worth noting that the basal level of the membrane oestrogen receptor GPR30 was higher in older mice within the epithelial layer, and ML385 treatment led to a downregulation of GPR30 in 6-month-old mice. In summary, ML385 decreases NRF2 TA in the colon and impacts its contractility and goblet cell numbers. Additionally, NRF2 TA influences the expression of oestrogen receptors in the colons of female mice.

## 1. Introduction

The process of moving, digesting, and absorbing food in the gastrointestinal system is essential for optimal nutrition and overall bodily function. The large intestine, as the final part of the digestive tract, facilitates the elimination of indigestible and unabsorbed food components while also absorbing water and electrolytes and producing vitamins. For the colon to function effectively, complex and well-coordinated communication between specialised cells is required, integrating neuronal and hormonal signals from different parts of the body.

Nuclear factor-erythroid 2-related factor-2 (NRF2), a protein encoded by the *NFE2L2* gene, is a transcription factor linked with cellular homeostasis that responds to stress. It affects the expression of antioxidant, cytoprotective, and detoxifying genes, such as heme oxygenase-1, NAD(P)H quinone dehydrogenase 1 (NQO1), or glutathione-disulfide reductase (GSR), in intestines [1] and other tissues [2]. Nevertheless, the multifaced role, especially in the onset and progression of several cancerous and non-cancerous diseases, should not be forgotten [3,4,5]. For instance, some studies suggested that the overactivation of NRF2 by suppression of its sequester Keap 1 (Kelch-like ECH-associated protein 1) leads to enhanced tumour survival by promoting antioxidant defences, metabolic reprogramming, and resistance to oxidative stress-induced cell death [6].

We previously showed that NRF2 transcriptional activity may influence colon function and structure in mice and that chemical inhibition of NRF2 transcriptional activity (TA) with ML385 (N-[4-[2,3-Dihydro-1-(2-methylbenzoyl)-1H-indol-5-yl]- 5-methyl-2-thiazolyl]-1,3-benzodioxole-5-acetamide) may change colon contractility in response to exogenous stimulation [7]. ML385 is a unique and selective NRF2 inhibitor. It interacts with NRF2 and alters the DNA-binding activity of the NRF2-MAFG (MAF BZIP Transcription Factor G) complex. ML385 has an IC50 of 1.9 µM and inhibits NRF2 transcriptional activity in a dose-dependent manner, with a maximal inhibitory concentration of 5 μM. The treatment of KEAP1 mutant H460 cells with ML385 results in a considerable reduction in NRF2 and a downstream of target gene expression. With the gain of NRF2 function, ML385 preferentially influences the colony-forming ability or proliferation of lung cancer cells [8].

Oestrogens are steroid hormones produced in all vertebrates, primarily in reproductive organs and, in lower quantities, in non-reproductive tissues, including the breast, liver, adrenal glands, adipose tissue, and gastrointestinal tract [9]. Oestrogen and its receptors have been shown to play a pathogenic role in conditions such as gastroesophageal reflux disease, oesophageal cancer, peptic ulcer disease, gastric cancer, irritable bowel syndrome, inflammatory bowel disease, and colon cancer [10,11,12]. Therefore, the control of oestrogen in the physiology of nonreproductive tissue is an emerging subject of research. The presence of absorptive and secretory epithelia among 17β-oestradiol (E2) target tissues highlights oestrogens’ essential role in regulating ion transport systems that maintain the body’s electrolyte and fluid balance [13,14]. Traditionally, it has been believed that E2 plays its role via a genetic process that combines oestrogen with nuclear oestrogen receptors and controls transcription [15,16,17] or by interacting with plasma membrane receptors [15,16,17], when it activates a variety of intracellular second messengers between seconds and minutes in various cells.

The distal colon has been identified as a target tissue for E2. Two intracellular receptors for E2, oestrogen receptor (ER)α and ERβ, have been discovered in colonic crypts [18]. Their function, as classical nuclear receptors and transcription factors, is sequestering transcriptional coactivators to regulate gene expression in the nucleoplasm. Additionally, G-protein coupled oestrogen receptor 1 (GPR30), a membrane-bound G-protein coupled receptor, has been suggested as an alternative oestrogen-binding protein, which adds another layer of complexity to oestrogen signalling [19,20]. It is the first alternative oestrogen receptor found molecularly [19,21], and it may be associated with the development and progression of inflammatory lesions in patients with Crohn’s disease [22].

Studies have shown that oestrogens inhibit gene transcription driven by the antioxidant response element (ARE) and facilitate cancer progression in an NRF2-dependent manner, independent of KEAP1-mediated NRF2 degradation. Immunoprecipitation assays further revealed a physical interaction between ERα and NRF2, mediated through the A/B (AF1) or C domains of NRF2 [23]. Therefore, oestrogen receptors might influence NRF2 transcriptional activity, but there is no published data on how chemical inhibition of NRF2 TA influences distal colon contractility.

Therefore, we aim to verify whether chemical inhibition of NRF2 transcriptional activity influences distal colon contraction in an age-dependent manner and affects oestrogen receptor signalling in female mice.

## 2. Results

**Figure 1:**
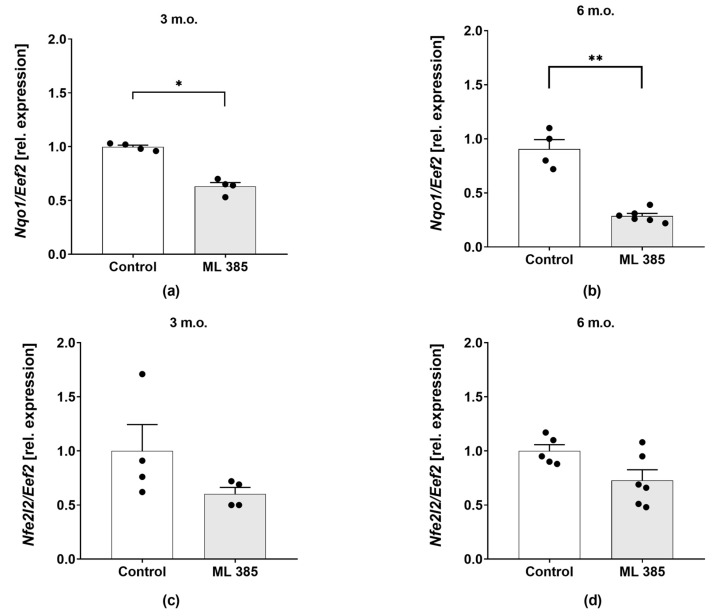
ML385 significantly inhibits Nrf2 transcriptional activity. Colon gene expression of *nqo1* (**a**) in 3 month-old, and (**b**) 6-month-old mice from the control and ML385 groups. Colon gene expression of *nfe2l2* (**c**) in 3 month-old, and, (**d**) 6-month-old mice from the control and ML385 groups. *Eef2*—a reference gene. Dots represent a single observation. N = 4–6 per group. * *p* < 0.05, ** *p* < 0.01, Mann–Whitney U or Kolmogorov–Smirnov test.

To confirm whether ML385 inhibits NRF2 transcriptional activity, we first assessed the expression levels of *nfe2l2* and NRF2-dependent gene *nqo1*. In young mice, we observed a significant downregulation of *nqo1* expression following ML385 treatment (*p* = 0.0286; Figure 1a, indicating reduced NRF2 activity. This effect was even more pronounced in older mice, where *Nqo1* expression was markedly decreased compared to the young animals (*p* = 0.0095; Figure 1b). In contrast, no significant changes were observed in the expression of *nfe2l2* (Figure 1c,d). These results collectively confirm that ML385 acts as an antagonist to NRF2 by specifically reducing its transcriptional activity.

**Figure 2:**
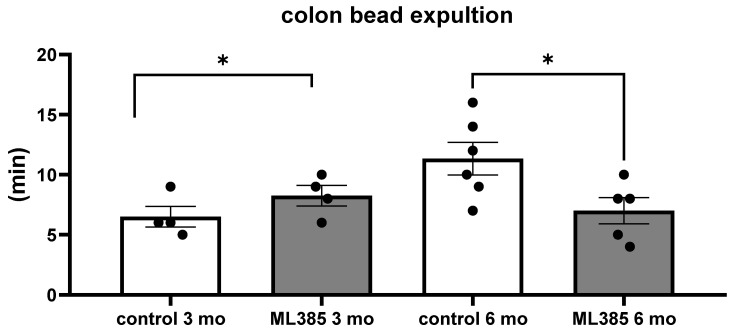
ML385 influences colon contractility in older mice. Colonic bead expulsion assay. Female control and ML385-treated mice, 3- and 6-month-old. N = 4–6 per group. Dots represent a single observation. * *p* < 0.05, ANOVA followed by Tukey’s test.

The in vivo colonic bead expulsion test was conducted to evaluate the functional impact of ML385 on colonic motility. Notably, the results revealed that only the older mice exhibited a measurable response to ML385 treatment. First, it was observed that the older mice had a significantly longer time to expel the beads compared to the younger mice, with a marked delay in expulsion (*p* = 0.0286; Figure 2). This suggests that in the absence of ML385, the colonic motility in older mice was slower, which may reflect age-related changes in gastrointestinal function.

However, upon administration of ML385, bead expulsion was significantly accelerated in the older mice (*p* = 0.039; Figure 2). The treated older mice exhibited a rate of bead expulsion that was comparable to that observed in the younger animals, indicating that ML385 effectively improved colonic motility in the ageing mice. These findings suggest that ML385 may restore or enhance colonic function in older animals by modulating NRF2 activity, which could have important implications for age-related gastrointestinal dysmotility. The lack of a similar response in the younger mice further emphasises that the observed effect of ML385 is specific to the older cohort, possibly due to age-related alterations in the NRF2 pathway or related signalling mechanisms.

**Figure 3:**
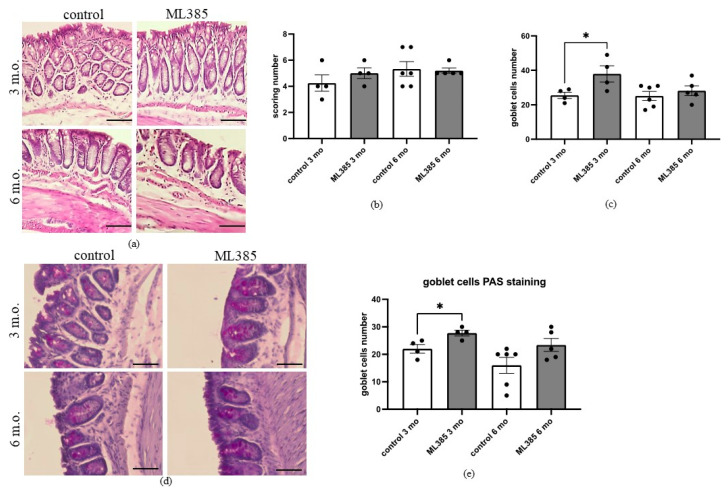
Inhibition of NRF22 transcriptional activity influences goblet cell number in young mice. (**a**) colon morphology (H&E staining), magnification 400×, (**b**) microscopic scoring, (**c**) goblet cells scoring, (**d**) colon morphology (PAS staining) magnification 400×, (**e**) goblet cells PAS scoring. Female control and ML385-treated mice, 3- and 6-month-old. N = 4–6 per group. Dots represent a single observation. * *p* < 0.05, ANOVA followed by Tukey’s test, scale bar 50 µm.

In the histological analysis of the colon (Figure 3a–e), we did not observe any significant differences between the 3-month-old and 6-month-old mice in terms of overall colon morphology or cellular composition. Both age groups exhibited similar structural features, suggesting that the natural ageing process did not result in obvious histological alterations within the time frame studied. However, a more detailed examination revealed a slight but significant increase in the number of goblet cells in young mice treated with ML385 (*p* = 0.0473; Figure 3c). Goblet cells, which are responsible for mucus secretion, play a key role in maintaining intestinal health, and their increased presence in response to ML385 suggests a potential compensatory mechanism in young mice.

To further validate this finding, we performed PAS staining, which confirmed the increase in goblet cell numbers in the young ML385-treated mice, with statistical significance observed (*p* = 0.0226; Figure 3e). In contrast, no significant differences in goblet cell counts were detected in the older mice, regardless of ML385 treatment. This lack of response in the older cohort may indicate that age-related changes in colonic physiology or cell signalling pathways could limit the ability of ML385 to modulate goblet cell numbers in the same manner as in the younger animals. These results suggest that while ML385 may have an impact on goblet cell numbers in young mice, its effects on cellular composition in the colon may be age-dependent, warranting further investigation into the underlying mechanisms.

**Figure 4:**
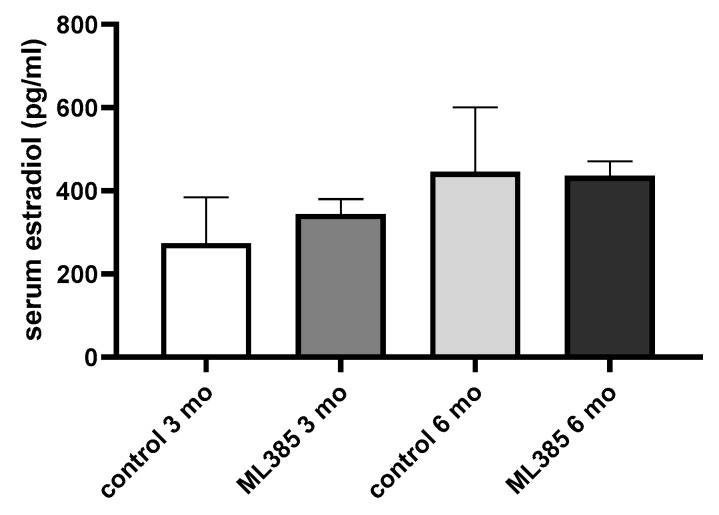
The influence of ML385 on serum oestrogen level. Female control and ML385-treated mice, 3 and 6-month-old. N = 4–6 per group. Dots represent a single observation. *p* values were calculated using an ANOVA followed by Tukey’s test.

To determine whether ML385 influences oestrogen signalling, we measured the serum levels of 17-β oestradiol (Figure 4) and found that chemical inhibition of NRF2 transcriptional activity did not affect oestradiol concentrations, regardless of the mice’s age. This indicates that ML385’s effects on colon physiology are not mediated by changes in circulating oestrogen levels.

**Figure 5:**
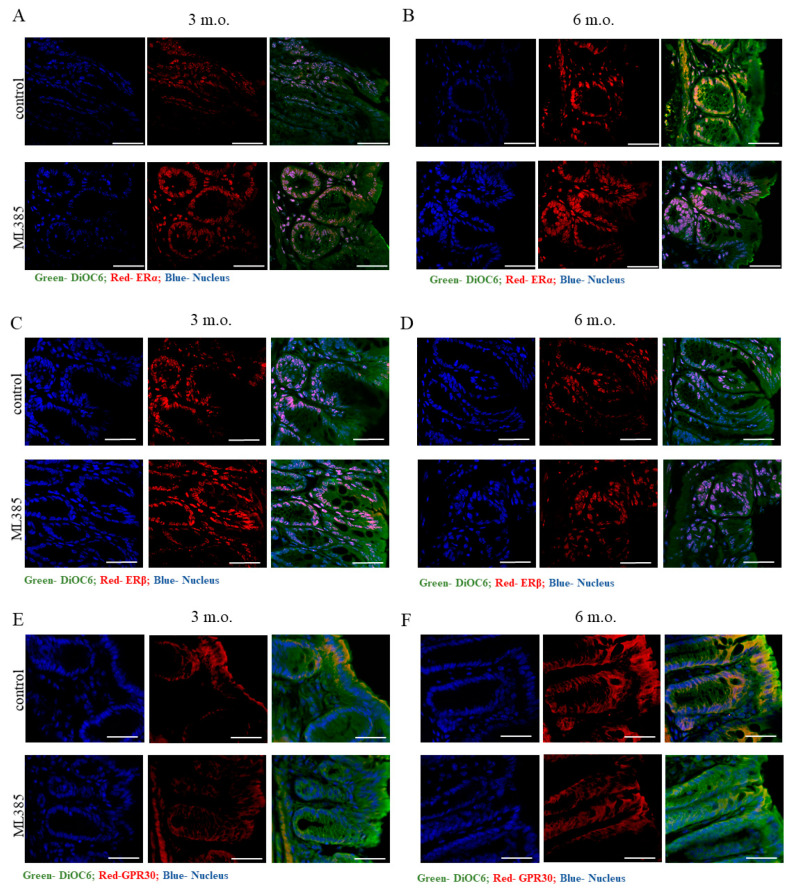
Effect of ML385 on oestrogen receptor localisation in the colon. Panels (**A**,**B**) show oestrogen receptor alpha (ERα, red), DiOC6 (green), and nuclei (blue); panels (**C**,**D**) show oestrogen receptor beta (ERβ, red), DiOC6 (green) and nuclei (blue); and panels (**E**,**F**) show GPR30 (red), DiOC6 (green), and nuclei (blue) in 3-month-old and 6-month-old mice treated with either vehicle or ML385. DiOC6-3,3′-Dihexyloxacarbocyanine Iodide cell membrane dye. Images were captured at 600× magnification, with a scale bar of 25 μm.

However, when we examined oestrogen receptor expression within the colon tissue using immunofluorescent staining, we observed an age-dependent pattern of ERα expression. In control mice, there was a notable decrease in ERα levels as the animals aged, with older mice showing significantly higher levels of ERα in colon tissues compared to their younger counterparts (Figure 5A,B). Interestingly, ML385 treatment appeared to counteract this age-related decline in ERα expression, leading to an increase in ERα levels, especially in the younger mice. This suggests that NRF2 inhibition may have a modulating effect on oestrogen receptor expression, particularly in the context of ageing.

In contrast, we found no significant changes in ERβ expression in response to either age or ML385 treatment (Figure 5C,D), indicating that ERβ may not be as responsive to changes in NRF2 activity or age-related shifts in oestrogen receptor regulation within the colon.

Of note, we observed an interesting trend in the expression of the membrane oestrogen receptor GPR30, which was found to be more highly expressed in the epithelial layer of older mice compared to young mice (Figure 5E,F). This may suggest that ageing may influence the localisation and abundance of GPR30 within colon tissue. Notably, ML385 treatment led to a downregulation of GPR30 expression, specifically in the older mice (Figure 5F), indicating that NRF2 inhibition may affect the distribution of membrane-bound oestrogen receptors in a manner that is age-dependent. Figure S1 presents negative controls of immunofluorescent staining for ERα, ERβ and GPER in the colon.

Taken together, these results suggest that chemical inhibition of NRF2 TA may modulate the localisation and expression of oestrogen receptors within the colon, with age playing a critical role in determining the extent and direction of these effects. The observed changes in ERα and GPR30 expression highlight potential age-specific alterations in oestrogen signalling pathways in the colon that are influenced by NRF2 inhibition.

## 3. Material and Methods

### 3.1. Animal Model

In this study, female C57BL/6J mice aged 3 and 6 months were used. The mice were kept in specific pathogen-free conditions in individually ventilated cages, maintained on a 14-h light/10-h dark cycle at a temperature of 22 ± 2 °C. They were given a standard diet and had ad libitum access to water. The experimental protocols received approval from the Ninth Local Ethics Committee for Animal Research in Lodz (Approval No. 59/ŁB 222/2021 from 6 December 2021). To block NRF2 transcriptional activity, the mice were administered 100 µL of ML385 (30 mg/kg/day; Tocris Bioscience, Bristol, UK) or 1% DMSO (Sigma-Aldrich, Dortmund, Germany) via intraperitoneal injection for 7 days prior to the isolation of colon tissue. The mice were divided into four groups: (1) control 3-month-old mice (n = 4), (2) ML385 3-month-old mice (n = 4), (3) control 6-month-old mice (n = 6), and (4) ML385 6-month-old mice (n = 6). After six days of treatment, the mice were anaesthetised with isoflurane; venous blood was collected for serum separation, and colon tissue was harvested for histological analysis (fixed in 10% formalin (POCH, Gliwice, Poland)) and gene expression analysis.

### 3.2. Colon Bead Expulsion Test

The colon bead expulsion test was employed to assess the speed of peristaltic waves in the colon. Mice were fasted for 12 to 14 h before the procedure. After fasting, the mice were lightly anaesthetised with 1% isoflurane, and a prewarmed glass bead (37 °C) was inserted 2.5 cm into the distal colon using a silicone feeding needle. The mice were then placed in individual cages on a white sheet, and the time taken for bead expulsion was recorded in minutes [7].

### 3.3. Serum Oestradiol Measurement

The level of colon 17-β-oestradiol was assessed using an ELISA with an R&D kit (KGE014; R&D Systems Europe, Hertfordshire, UK), following the manufacturer’s protocol for sample preparation and measurement. A 96-well plate was pre-coated with anti-oestradiol IgG. Samples and an oestradiol-horseradish peroxidase (HRP, from the kit) conjugate were added to the wells. After incubation, the wells were washed to remove any unbound material, and a substrate solution was introduced. The reaction was halted by adding Stop Solution, which ceased colour development and resulted in a colour change from blue to yellow. The intensity of the signal was inversely related to the amount of oestradiol in the sample, with absorbance measured at 450 nm.

### 3.4. Histological Staining and Analysis

Histological and immunofluorescent staining were conducted on frozen 10 μm tissue sections. Haematoxylin and eosin (H&E; Sigma, Dortmund, Germany) staining was used for general morphology, while periodic acid–Schiff (PAS, Abcam, Cambridge, UK) staining was applied to assess mucin, following previously described protocols [7,24]. The samples were examined under a light microscope (Olympus EP50, Olympus, Tokyo, Japan) using Firmware software (Olympus EP50, Japan) at a magnification of 400×. Three distinct tissue sections from the same sample were analysed for each assessment.

Goblet cells within the crypts were quantified using the area measurement tool in ImageJ software version 1.53t (Wayne Rasband, NIH, Bethesda, MD, USA). The results were presented as the percentage of goblet cells within the crypts.

The microscopic total damage score was assessed on H&E-stained slides using the following criteria described by [7]: goblet cell depletion (present = 1, absent = 0), crypt abscesses (present = 1, absent = 0), mucosal architecture integrity (normal = 1, moderate destruction = 2, extensive destruction = 3), muscle layer thickness (normal = 1, moderate thickening = 2, extensive thickening = 3), and cellular infiltration extent (normal = 1, moderate = 2, transmural = 3). Two researchers independently scored blinded samples, and the mean score for each sample was calculated.

For immunofluorescent staining, samples were permeabilised with 0.5% Triton X-100 for 20 min at room temperature (RT) and then blocked with 5% goat serum (Biowest, Nuaillé, France) in PBS containing 0.05% Tween 20 (Wash Buffer, WB; Sigma, Germany) for 1 h at RT. For quenching autofluorescence, the samples were incubated with Sudan Black B (Sigma, Germany) in 70% ethanol solution for 20 min at room temperature in the dark [25]. Following a wash in WB, samples were incubated overnight at 4 °C with primary antibodies diluted in 1% BSA in WB: rabbit anti-ERα polyclonal IgG (1:250; ab75635, Abcam, UK), rabbit anti-ERβ polyclonal IgG (1:250; ab3576, Abcam, UK), or rabbit anti-G-protein coupled receptor 30 (GPER) monoclonal IgG (ab260033, Abcam, UK). The next day, samples were washed again and incubated for 1 h at RT with secondary anti-rabbit antibodies conjugated to Alexa Fluor 568 (for ERα and ERβ) or Alexa Fluor 647 (for GPR30) at a 1:1000 dilution (IgG H+L, Life Technologies, Foster City, CA, USA). During a subsequent wash, nuclei were counterstained with Hoechst 33342 (1 μg/mL, Sigma-Aldrich, Germany). Imaging was performed with a meta-laser scanning confocal microscope (FV3000, Olympus, Japan) at 600× magnification, and data were analysed using ImageJ software (Wayne Rasband, NIH, USA).

### 3.5. Gene Expression Analysis

Trizol was used to extract RNA from sections of colon tissue measuring 0.5 centimetres in length. Using a High-Capacity cDNA Reverse Transcription Kit, cDNA was synthesised (Thermo Fisher Scientific, Waltham, MA, USA). RTqPCR was performed on Step-One Plus Real-Time PCR Devices utilising a Power SYBR^®^ Green PCR Master Mix as directed by the manufacturer (Thermo Fisher Scientific, Waltham, MA, USA). Primer sequences are presented in Table 1. Eukaryotic mouse translation elongation factor 2 (*Eef2*) was used as a reference gene. Relative gene expression was calculated using the ΔΔCt method.

### 3.6. Statistical Analysis

Data are expressed as mean ± SEM. For comparisons between two groups, Student’s *t*-test was used if the data were normally distributed; otherwise, the Mann–Whitney U or Kolmogorov–Smirnov test was applied. For comparisons involving more than two groups, ANOVA followed by Tukey’s post hoc test was used. The normality of the data was verified using the Shapiro–Wilk test. The Grubbs test identified statistically significant outliers, which were excluded from the analysis (conducted with GraphPad Prism 8.0 software). Statistical significance was set at *p* < 0.05.

## 4. Discussion

Our study showed that NRF2 transcriptional activity may be effectively inhibited by 7-day treatment of ML385 in female mice. Moreover, the effect is stronger in older mice. Previous studies showed that ML385 is a probe molecule that binds to NRF2 and suppresses the expression of its downstream target genes [8]. ML385 specifically binds to the Neh1 and Cap ‘N’ Collar Basic Leucine Zipper (CNC-bZIP) domains of NRF2, disrupting the interaction between the MAFG-NRF2 protein complex and regulatory DNA binding sequences. In the mouse model of ulcerative colitis, the combination of ML385 (30 mg/kg) with dextran sulphate sodium (DSS) significantly increased colon damage and withdrew the anti-inflammatory effect of tested drugs [26,27].

Our results also showed that NRF2 inhibition influences colon function (normalises speed of defecation) in older mice, which is in line with our previous studies on genetic knockouts of NRF2 [7]. Additionally, short-term pharmacological inhibition of NRF2 TA influenced colon morphology, as manifested in an increase in goblet cell numbers, especially in young mice. A similar observation was made by Kopacz et al. [28], who studied 4-day-old pups and embryos lacking NRF2 transcriptional activity (tKO) as well as their wild-type counterparts. They observed significant structural changes in the intestines of 4-day-old NRF2 tKO pups, including an elongated colon, altered crypt distribution, and an increase in goblet cell growth with substantially elevated levels of mucin 2.

It was shown that the delayed expulsion of colonic beads in NRF2 tKO animals could be due to a lack of ERβ ligand, and the function could be restored by administering oestradiol (E2) [7]. Our results suggest that chemical inhibition of NRF2 TA may affect nuclear ERα and cellular GPR30 receptor localisation within the colon tissue, and the effect may depend on age. Notably, both oestrogens and androgens influence the absorption of dietary lipids and lymphatic transport in the intestines [29,30]. In contrast, their roles in the regulation of colonic motility are inverse. While oestrogen encourages the relaxation of smooth muscle cells induced by nitric oxide [31,32], androgen stimulates their contraction via calcium sensitisers [33]. However, females may be more susceptible due to substantial fluctuations in sex hormones during the menstrual cycle, which are known to affect gastrointestinal function and stress sensitivity and are directly linked to the incidence and severity of inflammatory bowel syndrome [12,34]. Therefore, Kopacz et al. showed that functional impairment and pronounced microanatomical changes were observed only in female NRF2 tKO mice, highlighting the achievable role of oestrogens, which is in line with our results.

Chemical inhibition of NRF2 gives similar results to genetic inhibition of NRF2 in female mice [7]. Recent research demonstrates unequivocally that oestrogens and NRF2 are essential and interdependent factors in the maintenance of intestinal homeostasis [1,35]. The protective role of 17 β-oestradiol in various cell types can be attributed to the activation of NRF2 [36,37]; however, the activation of oestrogen receptors can inhibit NRF2 activity [38].

ERα and RXRα nuclear receptors physically interact with NRF2, form a protein–protein complex, and negatively regulate ARE gene expression, with ERα requiring the ligand oestrogen but RXRα not doing so. In contrast, downregulation of nuclear receptors, such as RXRα, has been documented in a variety of tumours, including non-small cell lung cancer [39,40,41]. Our data support the previous claims that NRF2 modifies the level of oestrogen receptors [7]. Under control conditions, oestrogen receptors are present in goblet cells and epithelial cells, and ERα is the main receptor in the male colon, followed by ERβ and GPER30 [32,42]. To determine which oestrogen receptor mediates the effect of E2 on NRF2 activation, Wu et al. evaluated NRF2 activity in the presence of selective ERα and ERβ antagonists, methyl-piperidino-pyrazole (MPP) [43] and 4-[2-Phenyl-5,7-bis(trifluoromethyl)pyrazolo[1,5-a]pyrimidin-3-yl]phenol (PHTPP), respectively [44]. The ERα antagonist MPP substantially inhibited E2-induced NRF2 activity. MPP also inhibited the synergistic effect of E2 and tert-butylhydroquinone (tBHQ) on NRF2 activation. In contrast, neither alone nor in combination with tBHQ, the ERβ antagonist PHTPP inhibited NRF2 induction by E2 interventions. These results indicate that ERα, but not ERβ, is required for E2-mediated activation of NRF2, at least in MCF7 cells. Importantly, Wu et al. demonstrated that oestrogen not only increased NRF2 activity by itself but also augmented the action of NRF2 activators (tBHQ) from 4 to 9 compared to cells treated with these compounds alone. This synergistic effect is consistent with the hypothesis that common NRF2 and E2 stimulants enhance NRF2 activity independently, with the former acting via KEAP1 and the latter via a PI3K/Akt/GSK3β-mediated pathway [45].

In addition, Cao et al. [46] discovered that the cholesterol metabolism intermediate dehydroepiandrosterone (DHEA) inhibits inflammation responses and barrier dysfunction in LPS-activated intestinal epithelial cells and that these effects are mediated by the GPR30-dependent activation of NRF2. DHEA inhibited NLRP3 inflammasome production by blockage of the p38-induced activation in LPS-stimulated macrophages and colon epithelial cells. Moreover, they discovered that oral DHEA treatment prevented colitis in rodents in a GPR30-dependent manner. These findings suggested that DHEA can be used as an effective anti-inflammatory agent for the prevention of colitis and that GPR30 may be a potential therapeutic target for inflammatory bowel disease [46].

ERβ signalling modifies colonic epithelial permeability in rat models and decreases ERβ mRNA expression, which was related to active colitis [47]. Studies showed that high expression of ERβ was related to a lower risk of colitis induced by DSS exclusively in female mice but not in male mice [48]. Recent patient data suggest a role for ERβ expression in the development and course of IBD, with one study showing that patients with active IBD had lower levels of ERβ receptors compared to healthy individuals and those in remission. However, this study did not take into account differences between patients due to age or gender, which may be important, especially in the context of oestrogen receptor signalling [49].

It should also be highlighted that NRF2 activity may be modulated by other signalling molecules which interact with ERs. For instance, NRF2 level may be regulated after activation of the aryl hydrocarbon receptor (AHR) and ERα loop in various tissues [23,50]. Another signalling factor which interacts with NRF2 and may be modulated by ERs is PPARγ (Peroxisome Proliferator-Activated Receptor Gamma). It has two ARE sequence presence in its promoter [51,52]. Of note, there is a putative PPRE sequence in the NRF2 promoter region [53]. Therefore, NRF2 activity may depend on different crosstalk, and further studies are needed to verify how they influence intestinal structure and function.

## 5. Conclusions

In conclusion, this study highlights several key findings: First, a 7-day intraperitoneal administration of ML385 to female mice is effective in significantly reducing NRF2 TA in the colon; Second, chemical inhibition of NRF2 TA impacts distal colon contractility in older female mice and alters the levels of goblet cells in younger females; Third, the inhibition of NRF2 TA may modulate the expression of ERs in the female colon.

These results suggest that NRF2 TA may play a critical role in regulating colon function and cellular composition and that its inhibition could have broader implications for oestrogen receptor expression and related physiological processes in the female colon.

The clinical relevance of this study may be associated with the interplay between NRF2 and oestrogen receptors on inflammatory bowel disease development and progression. Our previous study showed that patients with Crohn’s disease have lower levels of GPR30 receptor in inflamed colon areas [22]. Additionally, NRF2 level is higher in the colonic epithelium within inflamed areas [54]. Therefore, targeting ERs or NRF2 within the colon tissue, especially inflamed areas may be a potential aim in IBD treatment.

## Figures and Tables

**Table 1:** The primer sequences.

Gene Name	Primer Forward	Primer Reversed
*Nqo1*	5′-AGC GTT CGG TAT TAC GAT CC-3′	5′-AGT ACA ATC AGG GCT CTT CTC G-3′
*Nfe2l2*	5′-TCA CAC GAG ATG AGC TTA GG-3′	5′-TAC AGT TCT GGG CGG CGA CT-3′
*Eef2*	5′-GAC ATC ACC AAG GGT GT GCA-3′	5′-TCA GCA CAC TGG CAT ACA GG-3′

## Data Availability

The data used to support the findings of this study are available from the corresponding author upon request.

## Supplementary Materials

The following supporting information can be downloaded at: https://www.mdpi.com/article/10.3390/ijms252413647/s1.
