# Supplementary material for: Chemical Inhibition of NRF2 Transcriptional Activity Influences Colon Function and Oestrogen Receptor Expression in Mice at Different Ages
Source: Int J Mol Sci. 2024 Dec 20;25(24):13647. doi: 10.3390/ijms252413647 (PMC11678879; doi:10.3390/ijms252413647)
Supplement: Supplementary file 1 [file ijms-25-13647-s001.zip › ijms-3337024-supplementary.pdf]

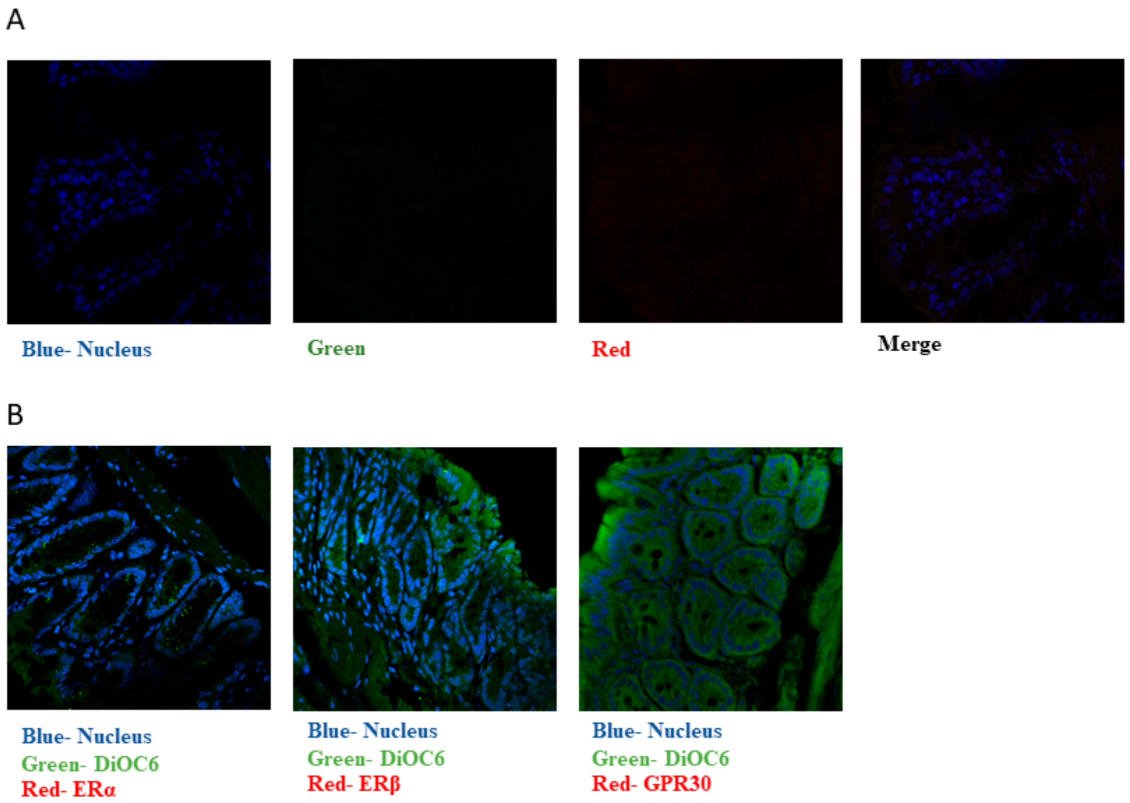

Figure S1: Negative controls of immunofluorescent staining for ERα, ERβ and GPER in the colon. (**A**) autofluorescence, (**B**) control without primary antibodies for tested receptors. Cell membrane dye-DiOC6- 3,3′-Dihexyloxacarbocyanine Iodide. Magnification 600×, scale bar 25 μm.
